# Resting-state functional connectivity in anterior cingulate cortex in normal aging

**DOI:** 10.3389/fnagi.2014.00280

**Published:** 2014-10-29

**Authors:** Weifang Cao, Cheng Luo, Bin Zhu, Dan Zhang, Li Dong, Jinnan Gong, Diankun Gong, Hui He, Shipeng Tu, Wenjie Yin, Jianfu Li, Huafu Chen, Dezhong Yao

**Affiliations:** ^1^The Key Laboratory for NeuroInformation of Ministry of Education, University of Electronic Science and Technology of ChinaChengdu, China; ^2^Radiology Department, Chengdu First People's HospitalChengdu, China

**Keywords:** aging, fMRI, resting state, functional connectivity, anterior cingulate cortex

## Abstract

Growing evidence suggests that normal aging is associated with cognitive decline and well-maintained emotional well-being. The anterior cingulate cortex (ACC) is an important brain region involved in emotional and cognitive processing. We investigated resting-state functional connectivity (FC) of two ACC subregions in 30 healthy older adults vs. 33 healthy younger adults, by parcellating into rostral (rACC) and dorsal (dACC) ACC based on clustering of FC profiles. Compared with younger adults, older adults demonstrated greater connection between rACC and anterior insula, suggesting that older adults recruit more proximal dACC brain regions connected with insula to maintain a salient response. Older adults also demonstrated increased FC between rACC and superior temporal gyrus and inferior frontal gyrus, decreased integration between rACC and default mode, and decreased dACC-hippocampal and dACC-thalamic connectivity. These altered FCs reflected rACC and dACC reorganization, and might be related to well emotion regulation and cognitive decline in older adults. Our findings provide further insight into potential functional substrates of emotional and cognitive alterations in the aging brain.

## Introduction

Age-related cognitive changes commonly occur with aging, especially in late life, and most often affect multi-domains involved in attention, working memory, and executive functioning, which have an impact on quality of life (Drachman, 1997; Hedden and Gabrieli, 2004; Grady, 2012). Although the neural mechanisms of neurodegenerative disorders remain somewhat elusive, these cognitive deficits combined with age accelerate the risk of dementia or Alzheimer's disease in older adults (Bishop et al., 2010). By contrast, accumulating evidence from functional neuroimaging and behavioral studies suggest that emotional function maintains well with aging, likely due to more available distribution of resource and reappraisal to emotion than in younger adults (Brassen et al., 2011; Mather, 2012; Suri and Gross, 2012). Previous researches have also revealed that many specific brain regions have specific involved emotional and cognitive processing. For example, prefrontal cortex is considered important for cognitive function, while the amygdala is critical to fear-related emotional processing (Ledoux, 2000; Koechlin et al., 2003). However, the neural mechanism underlying well-maintained emotional well-being and cognitive deficit in older adults remains unclear.

Functional association among distant brain regions of resting-state BOLD signals, which reflect intrinsically spontaneous brain activity, may provide a valid method to reveal age-related changes of cognitive and emotional processing (Ferreira and Busatto, 2013). More recently, functional neuroimaging studies have focused on the aging brain, and provide strong evidence of age differences in task- and resting-state (Grady, 2012; Ferreira and Busatto, 2013). Altered functional connectivity (FC) and heterogeneous changes of brain regions have also been observed with aging (Tomasi and Volkow, 2012; Wang et al., 2012). These alterations in FC have been found within and between networks, such as the default mode network and salience network (Onoda et al., 2012; Spreng and Schacter, 2012).

Convergent evidence from lesion and neuroimaging studies consistently indicate that the anterior cingulate cortex (ACC) is a key area involved in cognitive and emotional processing (Devinsky et al., 1995; Bush et al., 2000; Vytal and Hamann, 2010; Gasquoine, 2013). Altered FC of the ACC is also observed in aging and neuropsychiatric disorders, including schizophrenia (Yan et al., 2012) and major depressive disorder (Yoshimura et al., 2010; Korb et al., 2011). Using resting-state fMRI, Kelly and colleagues reported that patterns of FC in the ACC undergo an age-related shift from greater local FC to more a spatially limited pattern of FC from late childhood through adolescence to early adulthood (Kelly et al., 2009). Recently, several studies have also found that aging effects are vulnerable to long-range connections, leading to reduced anterior-posterior connectivity [e.g., the ACC/medial prefrontal cortex (MPFC)—posterior cingulate cortex (PCC)/precuneus] (Andrews-Hanna et al., 2007; Sambataro et al., 2010; Tomasi and Volkow, 2012). These changing in resting-state FC maps of the ACC might be associated with the alterations of emotion and cognition observed in older adults.

The ACC is typically subdivided into the rostral ACC (rACC) and dorsal ACC (dACC) based on their functional differentiation in emotional and cognitive processing (Bush et al., 2000; Allman et al., 2001). In line with the meta analysis (Vytal and Hamann, 2010), the rACC associated with emotion such as happiness and fear. In previous FC analyses (Torta and Cauda, 2011; Yu et al., 2011), the rACC mainly connects to the amygdala, orbitofrontal cortex (OFC), medial prefrontal cortex, superior temporal cortex, PCC/precuneus, insula and thalamus. These connections are involved in emotional processing, such as affective, motivational, and self-conscious emotion. The dACC, which is mainly related to the dorsolateral prefrontal cortex (DLPFC), dorsolateral parietal cortex, bilateral insula and some subcortical regions, plays an important role in cognitive control processes such as attention control (Davis et al., 2005), conflict monitoring (Botvinick et al., 2004; Weissman et al., 2005) and error detection (Holroyd et al., 2004; Polli et al., 2005). A variety of indirect observations have suggested roles for the rACC and dACC for emotional- and cognitive-related processing. However, no study has investigated whether the resting-state FC maps of the two subregions of ACC change with aging.

On the basis of previous functional neuroimaging and behavioral data, we hypothesized that maps of resting-state FC associated with two subregions of the ACC would undergo remodeling with aging to adapt to changes in cognition and emotion. To examine this hypothesis, we investigated the resting-state FC of the two ACC subregions in older adults compared with that in younger adults after ACC parcellation based on the similarity of FC profiles. Our findings might be helpful to investigate potential functional substrates of the emotional and cognitive alteration in the aging brain.

## Materials and methods

### Subjects

Forty-three right-handed healthy older adults and 37 healthy right-handed younger adults were recruited in this study. All participants had no history of substance abuse, neurological or psychiatric disorders. To eliminate the effect of inter-subject differences in behavioral measurements, all older subjects were assessed using neuropsychological test batteries including the Chinese 36-item short-form health survey (SF-36), which consisted of 36 items and tapped 8 health concepts (Li et al., 2003), and the Montreal Cognitive Assessment (MoCA), which was specifically developed in screening for mild cognitive impairment (Nasreddine et al., 2005). All participants received informed consent and the research protocol was approved by the Ethics Committee of the University of Electronic Science and Technology of China. All subjects were financially compensated for their time.

### Data acquisition

Subjects were scanned on a 3T MRI scanner (MR750; GE Discovery, Milwaukee, WI) in the MRI research center, University of Electronic Science and Technology of China. To minimize head motion, foam pads were used to fix their heads. Axial anatomical T1-weighted images were acquired using a 3-dimensional fast spoiled gradient echo (T1-3D FSPGR) sequence [repetition time (*TR*) = 6.008 ms, echo time (*TE*) = 1.984 ms, flap angle (*FA*) = 90, matrix = 256 × 256, field of view (FOV) = 25.6 × 25.6 cm^2^, slice thickness (no gap) = 1 mm] to generate 152 slices. Resting state functional MRI data were acquired using gradient-echo EPI sequences (*TR* = 2000 ms, *TE* = 30 ms, *FA* = 90°, matrix = 64 × 64, FOV = 24 × 24 cm^2^, slice thickness/gap = 4/0.4 mm, 32 slices per volume), with an eight channel-phased array head coil. All subjects underwent a 510 s resting state scanning to yield 255 volumes. To ensure magnetic field stabilization, the first five volumes were discarded. During data scanning, subjects were instructed to close their eyes without falling asleep, and not to think of anything in particular.

### Data preprocessing

#### Resting state fMRI data

Imaging data were conducted using the SPM8 software package (http://www.fil.ion.ucl.ac.uk/spm/). First, the data were corrected for the slice-timing and realigned for head motion correction. We excluded subjects whose head motion was more than 1 mm translation or 1° rotational movement during scanning. In addition, we also assessed translation and rotation in both groups using the following formula: head motion/rotation = $\frac{1}{M-1}\sum_{i\;=\;2}^{M}\sqrt{{\left|\Delta d_{x_{i}}\right|}^{2}+{\left|\Delta d_{y_{i}}\right|}^{2}+{\left|\Delta d_{z_{i}}\right|}^{2}}$, where *M* is the length of the time courses (*M* = 250 in this study); *x*_*i*_, *y*_*i*_ and *z*_*i*_ are translations/rotations at the *i*th time point in the *x*, *y*, and *z* directions, respectively, Δ*d*_*x*_*i*__ = *x*_*i*_ −*x*_*i* − 1_, and similar for *y*_*i*_ and *z*_*i*_. There were no significant differences between the two groups in head motion and rotation (two-sample two-tailed *t*-test, *T* = 1.50, *P* = 0.14 for translational motion, and *T* = 1.15, *P* = 0.25 for rotational motion). Second, functional data was coregistered to the native high-resolution anatomical images, then normalized to the Montreal Neurological Institute (MNI) template using a 12-parameter affine transformation and resampled to 3 × 3 × 3 mm^3^. Finally, the data were spatially smoothed with an 8-mm full-width at half maximum (FWHM) Gaussian kernel. Several nuisance covariates were also removed from the time course of all brain voxels using a multiple linear regression analysis. These covariates included six head motion parameters, global signal, write matter (WM) signal and cerebrospinal fluid (CSF) signal. The time courses were then examined by temporal band-pass filtering, which is a phase-insensitive filter between 0.01 and 0.08 Hz (Fox et al., 2005), to reduce the effects of low frequency drift and high frequency physiological noise.

#### T1-weighted images preprocessing

To correct for the effects of whole brain volume on functional connectivity analyses, T1-weighted images were analyzed through the voxel-based morphometry (VBM8 http://dbm.neuro.uni-jena.de/) toolbox in SPM8. In detail, T1-weighted images were normalized to MNI space using a diffeomorphic anatomical registration through exponentiated lie algebra (DARTEL) for each subject. The resulting images were then segmented into gray matter (GM), WM and CSF. Allowing for individual differences in brain size, the segmented images were modulated using the non-linear deformation. Individual gray matter volume (GMV) of the whole brain was calculated. A two-sample two-tailed *t*-test was used to assess the difference in GMV between the groups.

#### Functional parcellation analysis of ACC

We parcellated the ACC into two distinct subregions based on resting-state FC analyses. First, the region of interest (ROI) of the entire ACC was extracted according to the 82 anatomical structures using the AAL atlas (Tzourio-Mazoyer et al., 2002). The ROI was coregistered to subject-specific T1 space to correct for individual variation. The coregistered ROI was transformed into standard MNI space. Second, for a given subject, we created Pearson correlation maps of time courses between voxels in the entire ACC and voxels in whole brain. We then calculated cross-correlations for every voxel in the entire ACC according to the correlation maps; these cross-correlations reflect the spatial similarity of FC profiles between voxels in the entire ACC and voxels in whole brain. Third, we sorted the cross-correlation matrix using a *k*-means clustering algorithm. To avoid the effect of random initial cluster membership, the *k*-means algorithm was replicated 300 times and the solution with the minimal within-cluster variance was chosen. The choice of 2 clusters was initially based on previous studies, which suggested that there were two distinct subregions of the ACC (Bush et al., 2000). Finally, to define the rACC and dACC for each group, the group maps for clusters were determined by the 50% of the number of subjects that had a similar spatial clustering solution.

#### Analysis of resting-state data

Next, we exacted the mean signal for the two ROIs (rACC and dACC). FC analysis was performed by calculating Pearson correlation between the mean time course of ROIs and each voxel in the whole brain. A Fisher's r-to-z transformation was applied to convert correlation coefficients of each voxel to a normal distribution. Therefore, individual *Z*-score maps were created for each ROI and subject.

#### Statistical analysis

Statistical analysis of the functional correlations was performed in SPM8. First, to correct for the effects of atrophy on FC analyses, the whole brain GMV was regressed as a confounding covariate in the general linear model for each group. Then, the within-group *Z*-score map was analyzed with the random effect one-sample *t*-test. Statistical maps of significant connections with the rACC and dACC were created for each group. A *P*-value threshold of *P* < 0.05 (FDR-corrected, *k* = 23 adjacent voxels) was set to identify the significance level. Second, a two-sample *t*-test was performed with an explicit mask from the union set of the one-sample *t*-test results of the two groups. The significance threshold of group differences was set to *P* < 0.05 (FDR-corrected) and cluster size >23 adjacent voxels (621 mm^3^).

## Results

Forty three older adults and 37 younger adults were recruited in this study. Five and three older adults were excluded because of low MoCA score (<25) and poor performance of SF-36, respectively. Five older adults and four younger adults were excluded because of excessive head motion. Thirty older adults [age (mean ± *SD*): 51–76 years (62.4 ± 6 years), *n* = 17 females] and 33 younger adults [age (mean ± *SD*): 17–25 years (21.5 ± 2.8 years), *n* = 16 females)] were finally included in further functional connectivity analysis.

### GM volume

A significant decrease in whole GMV in older adults compared with younger adults was found (two-tailed *t*-test, *T* = 4.63, *P* = 1.4 × 10^−5^). Therefore, individual GMV of the whole brain was regressed out in the general linear model.

### Functional parcellation of the ACC

**Figure 1:**
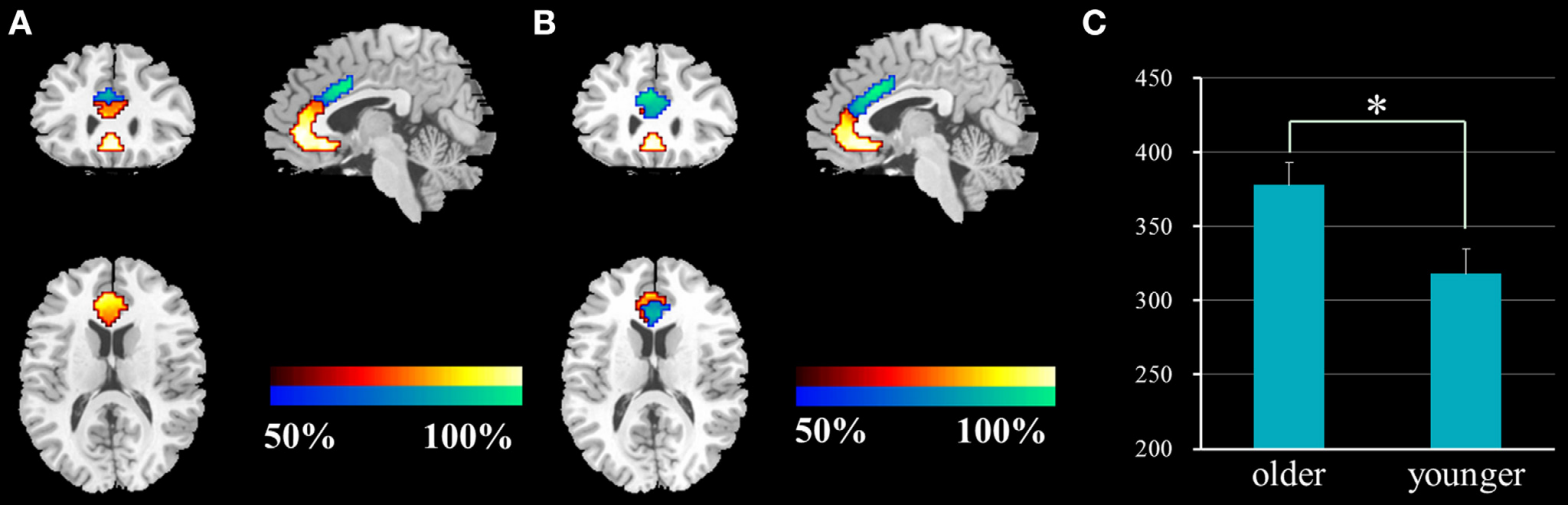
**Results of the functional parcellation analysis of ACC [rACC (red-yellow color gradient) and dACC (blue-green color gradient)] in younger adults (A) and older adults (B)**. Significant difference of voxel number in the dACC between the two groups **(C)**. ACC, anterior cingulate cortex; rACC, rostral ACC; dACC, dorsal ACC; ^*^P = 0.01.

The ACC was successfully parcellated into two distinct subregions for each group using the clustering algorithm based on spatial similarity of their FC profiles between voxel in ACC and voxels in the whole brain. The cluster solutions of the two groups are presented in Figure 1, which depicts the voxels that were classified similarly for approximately 50% of the subjects. Compared with younger adults, older adults demonstrated an increased number of voxels in the dACC and a decreased number of voxels in the rACC (two-sample two-tailed *t*-test, *T* = 2.66, *P* = 0.01).

### Resting-state functional connectivity

**Figure 2:**
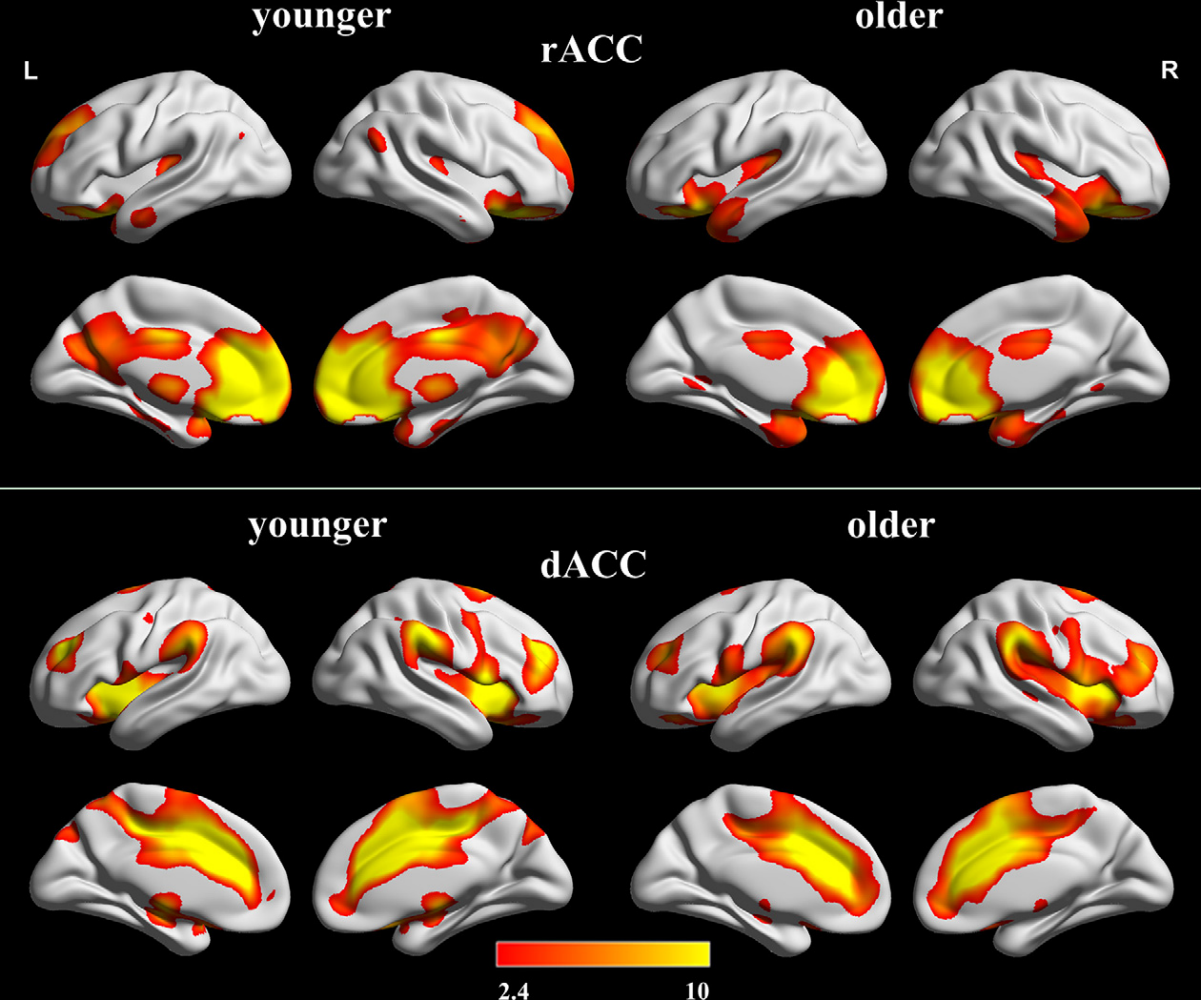
**Positive FC Maps of rACC and dACC in older and younger adults**. The statistical threshold was *P* < 0.05 (FDR-corrected, *k* = 23 adjacent voxels). Color bar indicates the *t*-value. FC, functional connectivity; rACC, rostral ACC; dACC, dorsal ACC.

The within-group FC maps seeded at the rACC and dACC (see Figure 2) were generated for each group, and the positive connectivity maps were only considered in further analysis.

#### FC map of rACC

**Figure 3:**
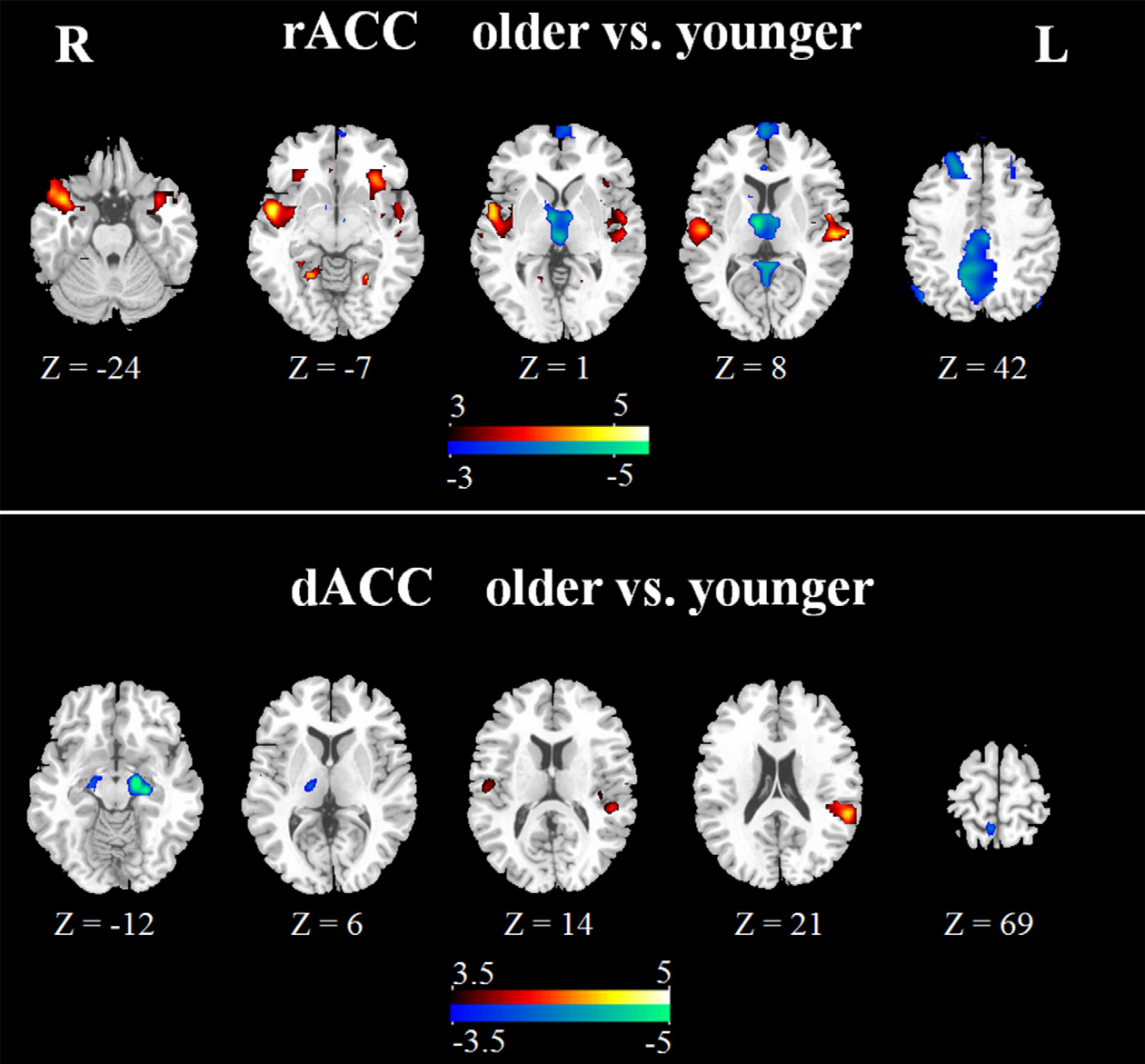
**Significant differences of rACC (upper) and dACC (lower) between the two groups**. Compared with younger adults, both increased (hot) and decreased (cool) connectivity was observed. The statistical threshold was *P* < 0.05 (FDR-corrected, *k* = 23 adjacent voxels).

In the younger group, the rACC was positively correlated with the superior/medial frontal gyrus, inferior frontal gyrus, superior temporal gyrus, medial temporal gyrus, posterior cingulate/ precuneus, hippocampus, amygdala, caudate and thalamus (Figure 2). In the older group, the rACC was positively correlated with the insula, inferior frontal gyrus, superior/medial frontal gyrus, putamen, superior temporal gyrus, superior frontal gyrus, hippocampus, and amygdala (Figure 2). Compared with younger adults, significantly increased connections were observed in the bilateral insula, superior temporal gyrus, inferior frontal gyrus, and putamen, while decreased connections were detected in the PCC/precuneus, superior/middle frontal gyrus, cingulate gyrus, thalamus and caudate in older adults (Figure 3, Table 1).

**Table 1:** **Significant differences for resting-state functional connectivity of the rACC and dACC in older adults compared with younger adults**.

**Regions**	**BA**	**MNI coordinates**			**Peak *T*-score**	**Cluster voxels**
		***x***	***y***	***z***		
**rACC**						
**Younger < older**						
Right superior temporal gyrus	BA 22	54	5	−5	5.64	1060
Right insula	BA 13	48	5	−14	4.58	
Right putamen		27	8	−14	4.2	
Left superior temporal gyrus	BA 41	−54	−19	7	4.92	848
Left inferior frontal gyrus	BA 47	−33	23	−11	4.47	
Left insula	BA 13	−48	2	−5	3.76	
Left putamen		−21	2	−14	3.33	
Left lingual gyrus	BA 19	−21	−61	−5	5.34	48
Right anterior cingulate gyrus	BA 24	3	29	−5	3.75	26
Right insual	BA 13	33	26	−11	3.65	66
**Younger > older**						
Right posterior cingulate	BA 29	6	−43	19	6.03	2318
Left posterior cingulate	BA 29	−6	−49	16	5.63	
Left precuneus	BA 30	−9	−64	31	5.30	
Right precuneus	BA 30	15	−58	37	4.99	
Left cingulate gyrus	BA 31	−3	−22	40	4.90	
Right thalamus		24	−28	13	4.35	
Right middle temporal gyrus	BA 39	39	−52	25	3.95	
Left anterior cingulate gyrus	BA 24	−3	29	16	6.26	2245
Right thalamus		9	−10	4	5.78	
Right medial frontal gyrus	BA 10	3	71	13	5.04	
Left thalamus						
Right superior frontal gyrus	BA 8	0	65	25	4.41	
Left superior frontal gyrus	BA 6	9	29	58	4.22	
Left medial frontal gyrus	BA 9	−3	62	34	4.21	
Left caudate		−18	17	16	3.83	
Right caudate		12	11	16	3.44	
Right anterior cingulate gyrus	BA 24	9	14	19	3.43	
Left middle temporal gyrus	BA 39	−42	−70	31	3.83	62
**dACC**						
**Younger < older**						
Left temporoparietal junction	BA 48	−54	−43	22	5.06	140
Right temporoparietal junction	BA 48	51	−25	−5	4.55	29
**Younger > older**						
Left parahippocampal gyrus	BA 34	−15	−13	−20	6.48	164
Right parahippocampal gyrus	BA 28	18	−13	−17	4.36	36
Right precuneus	BA 5	9	−52	67	4.15	46
Right thalamus		18	−19	4	4.07	43

BA, Brodmann area; dACC, dorsal ACC; rACC, rostral ACC.

#### FC map of dACC

Overall, the dACC was positively correlated with the superior frontal gyrus, cingulate gyrus, precentral gyrus, inferior parietal lobule, bilateral insula, precuneus, claustrum, temporoparietal junction (superior temporal gyrus and supramarginal gyrus) and thalamus in the two groups. Compared with younger adults, significantly increased connectivity was found in temporoparietal junction in older adults. Significantly decreased connectivity was detected in the hippocampus, right thalamus, and right precuneus (Figure 3, Table 1).

## Discussion

Accumulating evidence suggests that normal aging is associated with declining cognitive function and maintenance of emotional well-being. The ACC is a key area in cognitive and emotional processing (Devinsky et al., 1995; Bush et al., 2000; Gasquoine, 2013). In the present study, we used the clustering of resting-state FC profiles to parcellate the entire ACC into two subregions, and examined resting-state FC maps of the rACC and dACC in older adults. Relative to the younger adults, older adults demonstrated greater connection between the rACC and anterior insula, which usually connects with the dACC to respond to salient information. This suggests that older adults recruit more proximal brain regions of the dACC connected with insula to maintain salient responses. The rACC also showed increased FC with the superior temporal gyrus and inferior frontal gyrus in older adults, areas that are important for emotional regulation. Finally, older adults also demonstrated decreased integration between the rACC and default mode, as well as decreased dACC-hippocampal and -thalamic connectivity. These altered resting-state FCs might reflect a remodeling of function of the rACC and dACC with aging. This reorganization might be related to the alteration of emotional and cognitive function in older adults.

The rACC is involved in autonomic, visceromotor, and emotional regulation (Phillips et al., 2003). Previous FC studies have shown that the rACC is linked to the amygdala, OFC, MPFC, PCC/precuneus, insula, and superior temporal cortex (Yu et al., 2011). We observed increased FC between the rACC and bilateral insula in older adults relative to younger adults. The insula is involved in physiological, emotional feeling states and cognitive regulatory functions (Chang et al., 2013; Duerden et al., 2013), and the anterior insula and dACC are usually considered key components in response to varied forms of salience, including attention, pain and other homeostatic challenges (Seeley et al., 2007; Menon and Uddin, 2010). Consistent with previous reports, we also found FC between the dACC and anterior insula in the two groups. Therefore, we presumed that FC with the anterior insula included both the dACC and rACC in older adults. Using a relatively unbiased data-driven clustering of FC, which is commonly used in previous studies (Chang et al., 2013), we also found an increased number of voxels in the dACC and a decreased number of voxels in the rACC in older adults. Older adults showed increased connectivity between the dACC and bilateral anterior temporoparietal junction, which are major nodes in the salience network (Mars et al., 2012). Taken together, these findings might reflect the functional reorganization of brain in older adults, which meant that older adults might recruit more proximal brain regions of the dACC connected with the insula to maintain effective salient response.

In general, healthy older adults have well-maintained emotional well-being because of excellent avoidance of processing negative information (Mather, 2012). Here we observed significant increased FC between the rACC and the superior temporal gyrus, inferior frontal gyrus and putamen in older adults compared with younger adults. These regions are important for the production, appraisal and autonomic regulation of emotion (Phillips et al., 2003), and are activated during emotional processing (Winecoff et al., 2011). The superior temporal gyrus is considered a key brain region for multisensory integration and coupling of social and emotional responses to highly processed perceptual inputs (Hein and Knight, 2008). The inferior frontal plays a critical role in avoiding emotional interference (Shamay-Tsoory et al., 2009). In addition, more predominant activation was observed in inferior frontal gyrus in negative stimuli (sadness or anger) in contrast to positive stimuli (happiness) (Vytal and Hamann, 2010). The findings would be related to well regulation for negative emotion in older adults. Besides, the amygdala plays an important role in processing emotions and mediating fear responses (Hare et al., 2005; Vytal and Hamann, 2010). Previous resting state FC analysis has proven FC between amygdala and rACC in younger adults (Roy et al., 2009; Robinson et al., 2010). In this study, we found FC between rACC and amygdala in both groups, but no significant difference between groups was detected. It might mean that the functional integration between rACC and amygdala would preserve stable with aging. Thus, the present findings indicated that increased FC between the rACC and these emotion-related brain regions might underlie maintenance of emotional well-being in older adults.

Consistent with previous studies, we found a striking positive correlation between the rACC and PCC/precuneus, superior frontal gyrus and middle temporal gyrus in younger adults. These regions are involved in the default mode network (DMN), which is associated with internal-focused processes (Maddock et al., 2001; Spreng et al., 2009). However, older adults illustrated significantly decreased connectivity between the rACC and these regions of DMN. In some extent, rACC was also included in DMN, thus the findings suggested evidence of reduced integration of the default mode with aging. Our finding was consistent with the previous observation, in which older adults illustrated reduced FC within DMN associated with memory defect (Sambataro et al., 2010; Mevel et al., 2013). In addition, we also observed decreased connectivity between the dACC and bilateral hippocampal gyrus, right thalamus and right precuneus in older adults. The hippocampus is known to play a role in memory function (Burgess et al., 2002; Dickerson and Eichenbaum, 2010), while the precuneus is involved in visuo-spatial imagery, episodic memory retrieval and self-processing operations (Cavanna and Trimble, 2006). A number of studies have demonstrated memory deficits in aging (Olichney et al., 2010; McDaniel and Einstein, 2011; Persson et al., 2012; Park et al., 2013). In our study, the decreased FC found in the hippocampus, thalamus and precuneus might be associated with the deficit of working memory in aging. Our findings indicate that these decreased FCs might reflect defects of cognitive processing and executive behavior in aging.

Several limitations should be considered in this study. First, the number of the cluster *k* is not unique. *K* = 2 was used in this study because of the prior dichotomy of ACC function. It is also similar to that previously used to assess the emotional and cognitive processing function in older adults (Bush et al., 2000). Second, physiological noise should be considered, as the band-pass filtering of 0.01–0.08 Hz in the present study cannot eliminate respiratory and cardiac fluctuations completely, due to a relatively low sampling rate (*TR* = 2 s). Third, we removed WM, CSF, and global brain signals to remove confounding variance (Fox et al., 2009). Because of a controversy regarding global signal regression (Murphy et al., 2009), we also performed fMRI data analysis without global signal regression, and found that these two data preprocesses did not alter the major results in the present study.

In summary, we observed significantly altered resting-state FC maps of the rACC and dACC in older adults. The reorganization of the FC was indicative of better emotional well-being and cognitive decline in older adults. Moreover, the rACC might, at least in part, take part in the connection between the dACC and insula to maintain the salient response. Collectively, this study might be helpful to investigate the potential functional substrates of the emotional and cognitive alterations in the aging brain.

## Author contributions

Conceived and designed the work: Weifang Cao, Cheng Luo, and Dezhong Yao. Acquired the data: Dan Zhang, Jinnan Gong, Jianfu Li, Weifang Cao, Wenjie Yin, and Diankun Gong. Analyzed the data: Weifang Cao, Li Dong, Bin Zhu, Hui He, and Shipeng Tu. Wrote the paper: Weifang Cao, Cheng Luo, and Dezhong Yaof. All authors revised the work for important intellectual content. All of the authors have read and approved the manuscript.

### Conflict of interest statement

The authors declare that the research was conducted in the absence of any commercial or financial relationships that could be construed as a potential conflict of interest.
